# Dovitinib induces mitotic defects and activates the G_2_ DNA damage checkpoint

**DOI:** 10.1111/jcmm.12176

**Published:** 2013-11-18

**Authors:** Wing Yu Man, Joyce PY Mak, Randy YC Poon

**Affiliations:** a Division of Life Science Center for Cancer Research, and State Key Laboratory of Molecular Neuroscience, Hong Kong University of Science and TechnologyClear Water Bay, Hong Kong

**Keywords:** Checkpoint, DNA damage, mitosis, TKI258

## Abstract

Dovitinib (TKI258; formerly CHIR-258) is an orally bioavailable inhibitor of multiple receptor tyrosine kinases. Interestingly, Dovitinib triggered a G_2_/M arrest in cancer cell lines from diverse origins including HeLa, nasopharyngeal carcinoma, and hepatocellular carcinoma. Single-cell analysis revealed that Dovitinib promoted a delay in mitotic exit in a subset of cells, causing the cells to undergo mitotic slippage. Higher concentrations of Dovitinib induced a G_2_ arrest similar to the G_2_ DNA damage checkpoint. In support of this, DNA damage was triggered by Dovitinib as revealed by γ-H2AX and comet assays. The mitotic kinase CDK1 was found to be inactivated by phosphorylation in the presence of Dovitinib. Furthermore, the G_2_ arrest could be overcome by abrogation of the G_2_ DNA damage checkpoint using small molecule inhibitors of CHK1 and WEE1. Finally, Dovitinib-mediated G_2_ cell cycle arrest and subsequent cell death could be promoted after DNA damage repair was disrupted by inhibitors of poly(ADP-ribose) polymerases. These results are consistent with the recent finding that Dovitinib can also target topoisomerases. Collectively, these results suggest additional directions for use of Dovitinib, in particular with agents that target the DNA damage checkpoint.

## Introduction

Receptor tyrosine kinases (RTKs) are cell surface receptors for polypeptide growth factors, cytokines and hormones 1. A wide variety of cellular events, including cell growth, differentiation, apoptosis, mobility and angiogenesis are modulated by RTKs. As their dysregulation is implicated in the development and progression of many types of cancer, RTKs have become an important class of molecular targets for anti-cancer therapies 2.

Several small molecule inhibitors of RTKs in clinical use, such as Sorafenib and Sunitinib, in fact target multiple RTKs 3. The argument for the development of multi-target drugs for cancer treatments is that with the exception of a few malignancies that are driven by mutations of a single gene (such a BCR-ABL in chronic myeloid leukaemia), most cancers are caused by multiple events and involve more than one aberrant signalling pathway 4. Another rationale of using multi-target drugs is that drug resistance developed in cancer patients treated with single-target drugs is often because of the activation of alternative RTKs pathways.

Dovitinib (TKI258; formerly CHIR-258) is an orally bioavailable inhibitor of a number of RTKs 5. It targets several members of the class III, IV and V RTK family, including vascular endothelial growth factor receptor 1/2 (VEGFR1/2), fibroblast growth factor receptor 1/3 (FGFR1/3), and platelet-derived growth factor receptor beta (PDGFRβ). At least in cell line and animal models, Dovitinib is effective in haematological malignancies and solid tumours including acute myelogenous leukaemia 6, multiple myeloma 5, colon cancer 7, pancreatic cancer 8, hepatocellular carcinoma 9–10, renal cell carcinoma 11 and urothelial carcinoma 12. Clinical trials involving Dovitinib are being conducted for the treatment of haematological malignancies and solid tumours, including Phase III trials for metastatic renal cell carcinoma (ClinicalTrials.gov).

Interestingly, several studies have revealed that Dovitinib could induce cell cycle arrest. Huynh *et al*. found that Dovitinib triggers a G_2_/M cell cycle arrest in a hepatocellular carcinoma cell line 9. In contrast, treatment of cells from urothelial carcinoma 12 and lymphoplasmacytic lymphoma 13 appears to trigger an arrest in G_1_ phase. These results are somewhat surprising because the RTKs targeted by Dovitinib generally have not been associated with directly controlling the cell cycle engine.

In this study, we aimed to substantiate if Dovitinib indeed induces cell cycle delays. We found that Dovitinib triggered a G_2_ arrest in a variety of cancer cell lines. Dovitinib also promoted a delay in mitotic exit in a subset of cells. The G_2_ arrest was induced through the activation of the DNA damage checkpoint.

## Experimental procedures

### Cell culture

The HeLa used in this study was a clone that expressed the tTA tetracycline repressor chimera 14. Hep3B was obtained from the American Type Culture Collection (Manassas, VA, USA). Nasopharyngeal carcinoma (NPC) cell lines C666-1 15, CNE2 16, HNE1 17 and HONE1 17 were obtained from NPC AoE Cell Line Repository (The University of Hong Kong). No authentication was done by the authors. HeLa 18 and Hep3B 19 stably expressing histone H2B-GFP were used for live-cell imaging. A HONE1 cell line expressing histone H2B-mRFP was generated by infection of HONE1 cells with histone H2B-mRFP-expressing retroviruses (viruses were produced by co-transfection of histone H2B-mRFP in pREVTRE2 and VSV-G plasmids into Phoenix-gp cells 20) in the presence of 5 μg/ml of polybrene (Sigma-Aldrich, St. Louis, MO, USA). The transduced cells were selected with 200 μg/ml of hygromycin B (Life Technologies, Carlsbad, CA, USA) for ∼2 weeks before histone H2B-mRFP-expressing colonies were isolated. Cells were propagated in RPMI1640 (for C666-1) or DMEM (for other cell lines) supplemented with 10% (v/v) calf serum (Life Technologies; for HeLa) or foetal bovine serum (Life Technologies; for other cell lines) and 50 U/ml penicillin streptomycin (Life Technologies) in a humidified incubator at 37°C in 5% CO_2_. Unless stated otherwise, cells were treated with the following reagents at the indicated final concentration: Adriamycin (Sigma-Aldrich; 0.2 μg/ml), AZD7762 (Selleck Chemicals, Houston, TX, USA; 20 nM), MK-1775 (Selleck Chemicals; 250 nM), nocodazole (Sigma-Aldrich; 0.1 μg/ml), Olaparib (AZD2281; Selleck Chemicals; 1 μM) and UCN-01 (Sigma-Aldrich; 100 nM). Dovitinib was supplied by Novartis (Basel, Switzerland). Trypan blue analysis was performed as described 21. Cell-free extracts were prepared as described previously 22.

### Ionizing radiation

Ionizing radiation was delivered with a caesium^137^ source from a MDS Nordion Gammacell 1000 Elite Irradiator.

### Single cell gel electrophoresis assay

The method for detecting DNA breaks in individual cells was performed as described by Singh *et al*. 23 by using the pH >13 electrophoresis buffer, with the modification that GelRed Nucleic Acid Gel Stain (Biotium, Hayward, CA, USA; 1/1667) was used instead of ethidium bromide.

### Flow cytometry

Flow cytometry analysis after propidium iodide staining was performed as described previously 21.

### Live-cell imaging

The setup and conditions of time-lapse microscopy of living cells were as previously described 24.

### Bromodeoxyuridine incorporation assay

Bromodeoxyuridine (BrdU) incorporation assays were performed as described previously 25.

### Antibodies and immunological methods

Antibodies against β-actin 26, CDK1 27, and cyclin B1 18 were obtained from sources as described previously. Antibodies against CHK1, cyclin B2, phospho-histone H3^Ser10^ (Santa Cruz Biotechnology, Santa Cruz, CA, USA), phosphor-CDK1^Tyr15^, cleaved PARP1(Asp214; BD Biosciences, Franklin Lakes, NJ, USA), phosphor-CDK1^Thr161^ (Cell Signalling Technology, Beverly, MA, USA), phosphor-CHK1^Ser317^ (Abcam, Cambridge, UK) and phosphor-histone H2AX^Ser139^ (γ-H2AX; Bethyl Laboratory, Montgomery, TX, USA) were obtained from the indicated suppliers. Immunoblotting was performed as described 22.

### γ-H2AX staining

Cells grown on poly-L-lysine-treated coverslips were fixed by ice-cold methanol for 10 min. The cells were then washed twice with PBS, 5 min. each, before permeabilized and blocked with 3% bovine serum albumin (BSA) and 0.2% Tween-20 in PBS at 25°C for 30 min. The cells were washed twice with wash buffer (0.2% Tween-20 in PBS) before incubated with phosphor-histone H2AX^Ser139^ antibody (1/500 in 3% BSA, 0.2% Tween-20 in PBS) at 4°C for 16 hrs. The cells were then washed with wash buffer four times, for 5 min. each, and incubated in dark with Alexa Fluor 594 goat anti-rabbit IgG secondary antibodies (Life Technologies) at 25°C for 2 hrs. After washed four times with wash buffer for 5 min. each, the cells were stained with Hoechst 33342 (3 μg/ml in wash buffer) for 5 min., washed three times with wash buffer, before mounted with 100 mM N-propyl-gallate in 9:1 v/v glycerol:PBS.

## Results

### Dovitinib induces a G_2_ cell cycle arrest in different cancer cell lines

**Figure 1:**
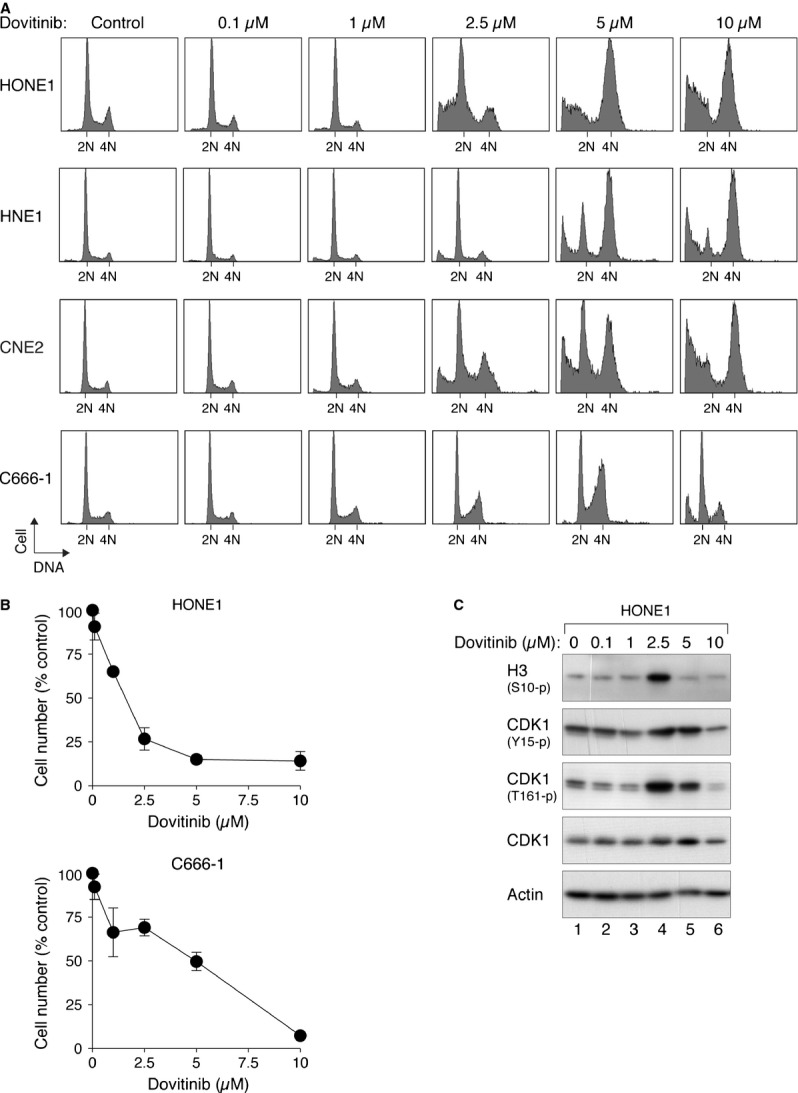
Dovitinib induces G_2_/M delay in nasopharyngeal carcinoma cell lines. (A) Dovitinib induces G_2_/M delay in a concentration-dependent manner. HONE1, HNE1, CNE2 and C666-1 (all originated from nasopharyngeal carcinoma) were exposed to the indicated concentrations of Dovitinib. After 48 hrs, the cells were harvested and analysed with flow cytometry. The positions of 2N and 4N DNA content are indicated. (B) Dovitinib reduces cell proliferation. HONE1 and C666-1 were treated with Dovitinib as in panel (A). The cells were then stained with trypan blue and the number of viable cells was analysed with a haemocytometer. Average ± SD from three independent experiments. (C) Dovitinib promotes an accumulation of mitotic cells. HONE1 cells were treated with different concentrations of Dovitinib as described in panel (A). Lysates were prepared and analysed with immunoblotting with antibodies that recognize the indicated proteins. Actin analysis was included to assess protein loading and transfer.

The effects of Dovitinib on the cell cycle were examined in cell lines from multiple types of cancers. Nasopharyngeal carcinoma is a rare but highly invasive cancer with effective chemotherapy still to be established. Four NPC cell lines (HONE1, HNE1, CNE2, and C666-1) were treated with different concentrations of Dovitinib. Flow cytometry analysis indicated that Dovitinib induced a G_2_/M arrest (4N DNA content) in a dose-dependent manner (Fig. 1A). A robust accumulation in G_2_/M was stimulated with 5 μM of Dovitinib in these cell lines. The increase of sub-G_1_ cells indicated that Dovitinib also triggered apoptosis at the higher concentrations. Accordingly, the apoptotic marker cleaved PARP1 also increased after Dovitinib treatment (see later). In agreement with the stimulation of G_2_/M cell cycle arrest and apoptosis, the number of viable cells, as measured directly using trypan blue exclusion analysis, was reduced by Dovitinib in a dose-dependent manner (Fig. 1B).

Unexpectedly, we found that histone H3^Ser10^ phosphorylation was increased after incubation with 2.5 μM of Dovitinib, and returning to basal level after incubation with higher concentrations of Dovitinib (Fig. 1C). Consistent with this, the activity of CDK1, as indicated by the activating phosphorylation (Thr161), was reduced after treatment with 5–10 μM of Dovitinib. These data suggested that Dovitinib could both stimulate mitosis and induce G_2_ arrest, both of which are consistent with the 4N DNA contents observed from the flow cytometry analysis. Moreover, the same DNA content could also be resulted from mitotic slippage, essentially generating G_1_ cells with tetraploid DNA content 28.

**Figure 2:**
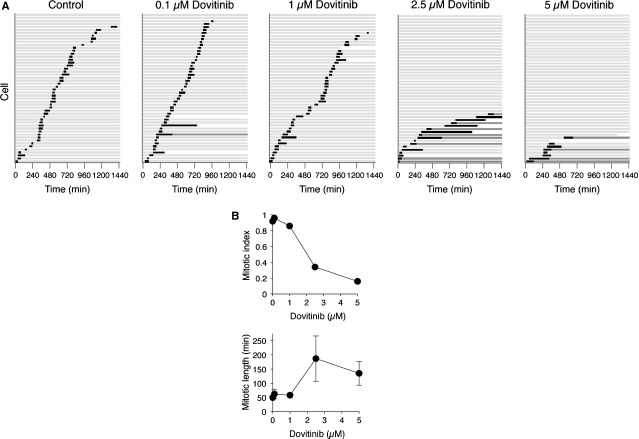
Dovitinib promotes mitotic defects and G_2_ arrest. (A) Different concentrations of Dovitinib induce G_2_ arrest or mitotic slippage. HONE1 expressing histone H2B-mRFP was incubated with the indicated concentrations of Dovitinib. Individual cells were then tracked for 24 hrs with time-lapse microscopy. Each horizontal bar represents one cell (*n* = 50). Key: light grey=interphase; black=mitosis (from DNA condensation to anaphase or mitotic slippage); dark grey=interphase after mitotic slippage; truncated bars=cell death. (B) The mitotic index and the duration of mitosis (mean ± 90% confidence interval) during the imaging period were quantified.

To distinguish these possibilities, we next analysed Dovitinib-treated cells dynamically using time-lapse microscopy. HONE1 stably expressing a histone H2B-mRFP was generated and subjected to live-cell imaging to track individual cells. As expected, control cells underwent mitosis randomly over the 24 hrs imaging period (Fig. 2A). In agreement with the flow cytometry analysis, the cell cycle was not significantly affected by 0.1–1 μM of Dovitinib. In contrast, higher concentrations of Dovitinib prevented cells from entering mitosis (quantified in Fig. 2B). Notably, for cells that could enter mitosis, Dovitinib also induced defects in mitotic exit. We will address this mitotic delay in more detail in the next section.

**Figure 3:**
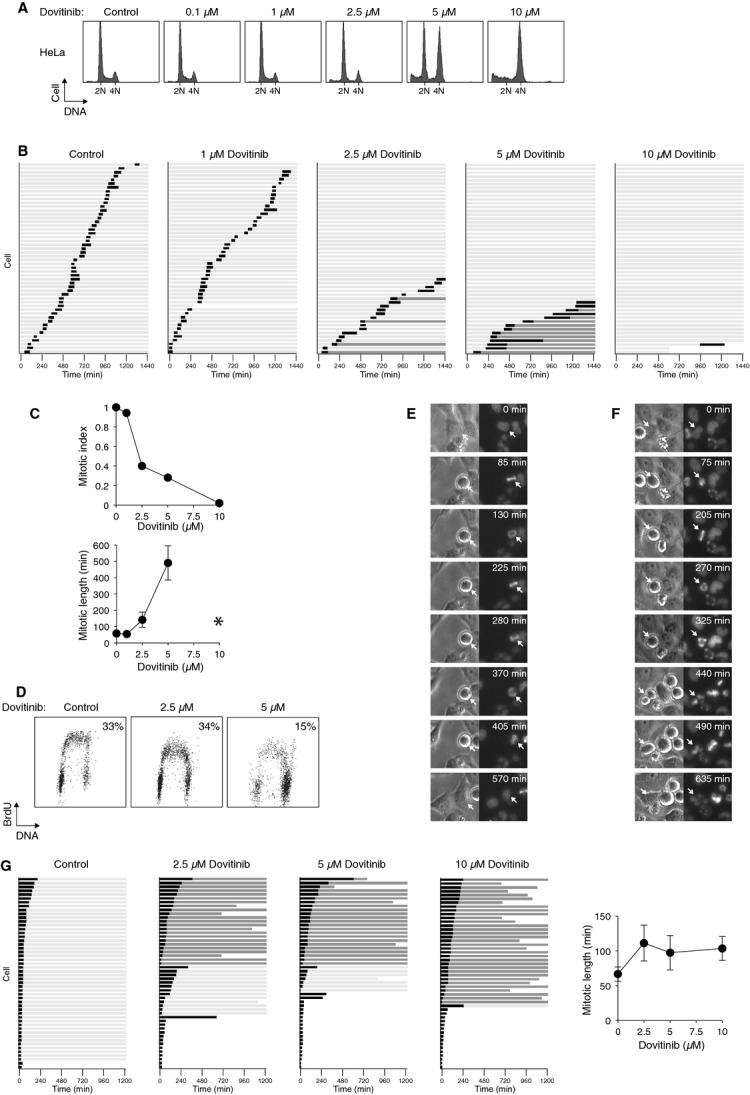
Dovitinib induces mitotic exit defects and G_2_ arrest in HeLa cells. (A) Dovitinib induces G_2_/M arrest in a concentration-dependent manner. HeLa cells were exposed to the indicated concentrations of Dovitinib. After 24 hrs, the cells were harvested and analysed with flow cytometry. The positions of 2N and 4N DNA content are indicated. (B) Dovitinib induces G_2_ arrest or mitotic slippage. HeLa expressing histone H2B-GFP was incubated with the indicated concentrations of Dovitinib. Individual cells were then tracked for 24 hrs with time-lapse microscopy. Each horizontal bar represents one cell (*n* = 50). Key is the same as in Figure 2A. (C) Different concentrations of Dovitinib induces G_2_ arrest or mitotic slippage. Cells were imaged as described in panel (B). The mitotic index and the duration of mitosis (mean ± 90% confidence interval) during the imaging period were quantified. Asterisk: because of the low number of mitotic cells, the mitotic duration after treatment with 10 μM of Dovitinib was not quantified. (D) Dovitinib inhibits DNA synthesis. HeLa cells were incubated with buffer or the indicated concentrations of Dovitinib. After 23 hrs, the cells were pulsed with BrdU for 1 hrs before BrdU incorporation was analysed with flow cytometry. The percentage of BrdU-positive cells is indicated. (E) Dovitinib triggers mitotic slippage. HeLa expressing histone H2B-GFP was incubated with Dovitinib (5 μM) and subjected to live-cell imaging. Still images (bright field and GFP) of a representative cell (arrow) that underwent mitotic slippage are shown. (F) Cytokinesis failure in Dovitinib-treated cells. HeLa expressing histone H2B-GFP was incubated with Dovitinib (5 μM) and subjected to live-cell imaging. Still images (bright field and GFP) of a representative cell (arrow) that underwent cytokinesis failure are shown. (G) Dovitinib induces mitotic exit defects in cells released from an early mitotic block. HeLa expressing histone H2B-GFP was incubated with nocodazole for 8 hrs. Mitotic cells were isolated by mechanical shake off. The nocodazole was removed by extensive washing and replated in fresh medium containing the indicated concentrations of Dovitinib. Individual cells were then tracked for 20 hrs with time-lapse microscopy. Each horizontal bar represents one cell (*n* = 50). Key is the same as in Figure 2A. The duration of mitosis (mean ± 90% confidence interval) was quantified.

**Figure 4:**
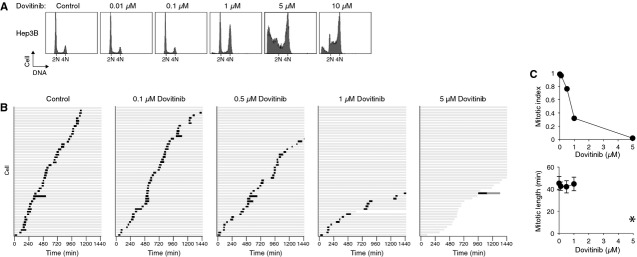
Dovitinib induces G_2_ arrest but not mitotic defects in Hep3B cells. (A) Hep3B cells display 4N DNA content after Dovitinib treatment. Hep3B cells were incubated with the indicated concentrations of Dovitinib. After 24 hrs, the cells were harvested and analysed with flow cytometry. The positions of 2N and 4N DNA content are indicated. (B) Dovitinib induces G_2_ arrest and cell death in Hep3B in a concentration-dependent manner. Hep3B expressing histone H2B-GFP was incubated with the indicated concentrations of Dovitinib. Individual cells were then tracked for 24 hrs with time-lapse microscopy. Each horizontal bar represents one cell (*n* = 50). Key is the same as in Figure 2A. (C) Dovitinib does not affect mitotic exit in Hep3B. Hep3B cells were treated and imaged as described in panel (B). The mitotic index and the duration of mitosis (mean ± 90% confidence interval) during the imaging period were quantified. Asterisk: because of the low number of mitotic cells, the mitotic duration after treatment with 5 μM of Dovitinib was not quantified.

To ensure that the G_2_ cell cycle arrest was not restricted to NPC cell lines, we also investigated the responses of HeLa cells to Dovitinib. Figure 3A shows that Dovitinib also induced a G_2_/M delay in HeLa cells. Single-cell analysis revealed that while most cells exhibited an interphase arrest (Fig. 3B), a subset of cells underwent a protracted and defective mitosis (see below). Entry into mitosis over the 24 hrs period was reduced to ∼40% in the presence of 2.5 μM of Dovitinib (Fig. 3C). Finally, a G_2_ cell cycle was also induced by Dovitinib in Hep3B, a cell line from hepatocellular carcinoma origin (see Fig. 4 below).

As a population of cells containing G_1_ DNA content was present after exposure to Dovitinib (in particular 2.5 μM, Figs 1A and 3A), we next examined if entry into S phase was perturbed. Figure 3D shows that BrdU incorporation was not affected following treatment with 2.5 μM of Dovitinib. The lower level of BrdU incorporation at 5 μM of Dovitinib simply reflected the lower percentage of G_1_ cells. These results indicated that Dovitinib did not significantly affect G_1_-S transition. Taken together, these results indicated that at micro-molar range, Dovitinib induces a G_2_ cell cycle arrest in different cancer cell lines.

### Dovitinib induces defective mitotic exit and promotes mitotic slippage

In addition to inducing a G_2_ phase arrest, treatment of HONE1 cells with 2.5–5 μM of Dovitinib also resulted in mitosis more protracted than in control cells (Fig. 2B). This is consistent with the increase in histone H3^Ser10^ phosphorylation (Fig. 1C). Furthermore, many of the cells were unable to exit mitosis properly and underwent mitotic slippage, resulting in a single daughter cell (Fig. 2A).

Similar results were obtained with Dovitinib-treated HeLa cells. Increasing concentrations of Dovitinib caused a decrease in mitotic entry and an increase in the duration of mitosis of the remaining cells (Fig. 3C). While the duration from DNA condensation to anaphase was ∼50 min. in control cells, this was extended to ∼500 min. in Dovitinib-treated cells. All the cells exited mitosis with mitotic slippage after treatment with 5 μM of Dovitinib (Fig. 3B).

Dovitinib-treated cells could form a metaphase plate similarly as control cells (data not shown), suggesting that early events of mitosis were not affected. However, the metaphase cells were unable to proceed to anaphase properly, indicating that the extension of mitosis was mainly because of a delay from metaphase to anaphase. The DNA eventually decondensed either without a proper anaphase (an example is shown in Fig. 3E) or with defective anaphase that resulted in cytokinesis failure (an example is shown in Fig. 3F).

As mitotic entry was prevented by the Dovitinib-mediated G_2_ arrest, we next studied the defective mitosis in more detail using a strategy that circumvented the G_2_ arrest. Cells were first trapped in early mitosis using nocodazole before released into fresh medium containing Dovitinib (Fig. 3G). As expected, control cells exited mitosis normally with anaphase and cytokinesis. By contrast, mitotic slippage occurred in cells treated with Dovitinib after a relatively long mitosis.

Notably, although all cell types we examined were arrested at G_2_/M after Dovitinib treatment, not all of them displayed defective mitosis. Flow cytometry analysis indicated that exposure to Dovitinib triggered a G_2_/M arrest in Hep3B cells (Fig. 4A). Live-cell imaging revealed that the mitotic index was reduced in a dose-dependent manner (Fig. 4B and C). Unlike HONE1 and HeLa cells, however, the length of mitosis was not affected in Hep3B. Instead, a large portion of cells underwent apoptosis directly (Fig. 4B). Collectively, these results indicate that a portion of Dovitinib-treated cells were unable to undergo anaphase properly, resulting in mitotic slippage.

### Dovitinib induces DNA damage and promotes inhibitory phosphorylation of CDK1

**Figure 5:**
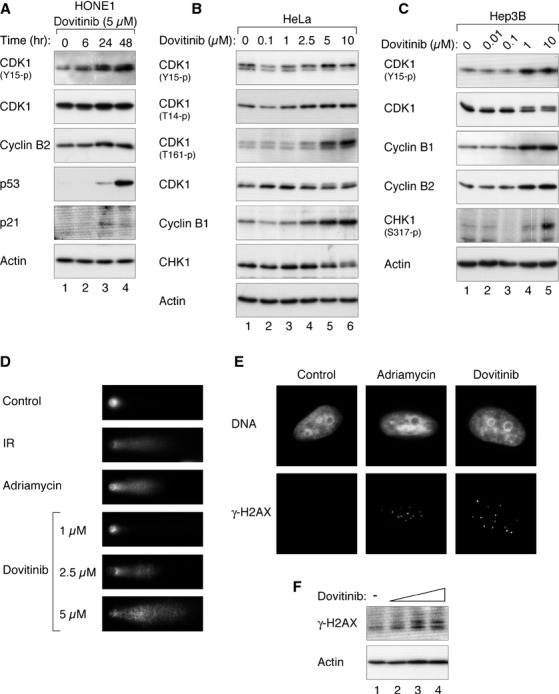
Dovitinib activates the G_2_ DNA damage checkpoint. (A) Dovitinib induces phosphorylation of CDK1^Tyr15^ in nasopharyngeal carcinoma cells. HONE1 cells were incubated with Dovitinib (5 μM) and harvested at the indicated time-points. Lysates were prepared and the indicated proteins were detected with immunoblotting. Equal loading of lysates was confirmed by immunoblotting for actin. (B) Dovitinib induces phosphorylation of CDK1^Tyr15^ in HeLa cells. HeLa cells were exposed to the indicated concentrations of Dovitinib for 24 hrs. Lysates were prepared and analysed with immunoblotting. Equal loading of extracts was accessed by immunoblotting of actin. (C) Dovitinib induces phosphorylation of CDK1^Tyr15^ in Hep3B cells. Hep3B cells were treated with the indicated concentrations of Dovitinib for 24 hrs. Lysates were prepared for immunoblotting analysis. Actin was probed to confirm equal loading. (D) Dovitinib induces DNA damage. HeLa cells were treated with buffer or the indicated concentrations of Dovitinib. After 36 hrs, the cells were processed for single cell gel electrophoresis assay. As positive controls, cells were also treated with ionizing radiation (IR, 10 Gy) and Adriamycin and harvested after 1 and 36 hrs respectively. Representative images from *n* = 50 are shown. (E) Dovitinib promotes the formation of γ-H2AX foci. HeLa cells were treated with buffer or 5 μM of Dovitinib. After 24 hrs, the cells were fixed and stained with antibodies against γ-H2AX and the DNA counterstained with Hoechst 33342. As a positive control, cells were treated with Adriamycin and harvested after 7 hrs. Representative images from at least 100 cells are shown. (F) Dovitinib increases γ-H2AX. HeLa cells were incubated with buffer or increasing concentrations of Dovitinib (1, 2.5, and 5 μM). After 24 hrs, the cells were harvested and analysed with immunoblotting. Actin analysis was included to assess protein loading and transfer.

As part of the effects of Dovitinib resemble that of DNA damage, we next investigated if the G_2_ DNA damage checkpoint is activated after Dovitinib challenge. Figure 5A shows that exposure of HONE1 cells to Dovitinib resulted in an increase in CDK1^Tyr15^ phosphorylation. Interestingly, p53 was also activated by treatment with Dovitinib. However, the fact that HONE1 cells were arrested mainly at G_2_ phase (Fig. 1A) suggested that the p53-dependent G_1_ DNA damage checkpoint may not be as effective as the G_2_ DNA damage checkpoint in causing a cell cycle arrest.

Dovitinib also promoted CDK1^Tyr15^ phosphorylation in HeLa cells (Fig. 5B). Phosphorylation of another inhibitory site of CDK1 (Thr14) was similarly increased. Although Dovitinib promoted the accumulation of cyclin B and the phosphorylation of the activating T-loop (Thr161; because of the accumulation of G_2_ cells), these results indicated CDK1 was inactivated by inhibitory phosphorylation.

Similar results were obtained with Hep3B cells (Fig. 5C). Thus, Dovitinib promoted CDK1 inhibitory phosphorylation in cell lines with different p53 background: HONE1, functional p53 29; HeLa, non-functional p53 30; and Hep3B, p53-null 31. The activation of CHK1, as seen with gel mobility shifts (Fig. 5B) or a phosphor-CHK1^Ser317^-specific antibody (Fig. 5C), agreed with the activation of the G_2_ DNA damage checkpoint.

To see directly if Dovitinib could induce DNA damage, we analysed whether DNA breaks were generated by single cell gel electrophoresis assay (comet assay). Figure 5D shows that DNA damage was induced by Dovitinib similarly as DNA damaging agents including ionizing radiation and Adriamycin. We also use phosphorylation of histone H2AX (γ-H2AX) as an indicator of DNA double-stranded breaks 32. An increase of γ-H2AX foci as detected with immunostaining (Fig. 5E) or γ-H2AX signal as detected with immunoblotting (Fig. 5F) was induced by Dovitinib.

Taken together, these data indicate that the Dovitinib-mediated G_2_ cell cycle arrest was associated with an activation of the DNA damage checkpoint and an increase in CDK1 inhibitory phosphorylation.

### Dovitinib-mediated G_2_ arrest can be abrogated by disrupting the DNA damage checkpoint

**Figure 6:**
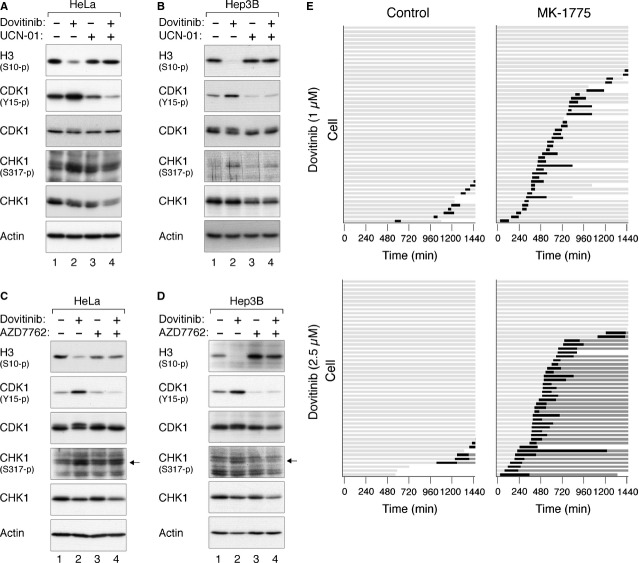
Dovitinib-mediated G_2_ arrest can be abrogated by disrupting the DNA damage checkpoint. (A) Dovitinib-activated checkpoint can be abrogated with UCN-01 in HeLa cells. HeLa cells were incubated with Dovitinib (2.5 μM) for 16 hrs. UCN-01 was then added for a further 8 hrs. Nocodazole was also added at the same time to trap cells in mitosis. Lysates were prepared and the indicated proteins were detected by immunoblotting. (B) Dovitinib-activated checkpoint can be abrogated with UCN-01 in Hep3B cells. Hep3B cells were incubated with Dovitinib (1 μM) for 16 hrs. UCN-01 was then added for a further 8 hrs. Nocodazole was also added at the same time to trap cells in mitosis. Lysates were prepared and the indicated proteins were detected by immunoblotting. (C) Dovitinib-activated checkpoint can be abrogated by CHK1 inhibition in HeLa cells. HeLa cells were incubated with Dovitinib (2.5 μM) for 16 hrs. AZD7762 was then added for a further 8 hrs. Nocodazole was also added at the same time to trap cells in mitosis. Lysates were prepared and the indicated proteins were detected by immunoblotting. (D) Dovitinib-activated checkpoint can be abrogated by CHK1 inhibition in Hep3B cells. Hep3B cells were incubated with Dovitinib (1 μM) for 16 hrs. AZD7762 was then added for a further 8 hrs. Nocodazole was also added at the same time to trap cells in mitosis. Lysates were prepared and the indicated proteins were detected by immunoblotting. (E) Dovitinib-activated checkpoint can be abrogated with a WEE1 inhibitor. Hep3B expressing histone H2B-GFP were incubated with the indicated concentrations of Dovitinib for 16 hrs. Control buffer or MK-1775 was added just before imaging. Individual cells were then tracked for 24 hrs with time-lapse microscopy. Each horizontal bar represents one cell (*n* = 50). Key is as the same as Figure 2A.

To test the hypothesis that Dovitinib activates the G_2_ DNA damage checkpoint, we next examined if small molecule inhibitors of the checkpoint could overcome the Dovitinib-mediated arrest. A CHK1 inhibitor UCN-01 33 was initially used to disrupt the checkpoint. As expected, Dovitinib stimulated the phosphorylation of CDK1^Tyr15^, thereby preventing cells from entering into mitosis (as indicated by the decrease in histone H3^Ser10^ phosphorylation). Addition of UCN-01 reversed the phosphorylation of CDK1^Tyr15^ and stimulated mitotic entry in both HeLa (Fig. 6A) and Hep3B (Fig. 6B) cells, indicating that CHK1 inhibition could abrogate the Dovitinib-mediated G_2_ arrest. Similar uncoupling of the Dovitinib-mediated G_2_ arrest was induced by a more specific CHK1 inhibitor AZD7762 34 in both HeLa (Fig. 6C) and Hep3B (Fig. 6D) cells.

We also examined if inhibition of WEE1 (a downstream target of CHK1) could abrogate Dovitinib-mediated arrest. A WEE1 inhibitor MK-1775 35 was added to Dovitinib-treated Hep3B cells followed by tracking with time-lapse microscopy (Fig. 6E). As expected, Dovitinib (1 and 2.5 μM) induced a cell cycle arrest in Hep3B cells. Addition of MK-1775 promoted mitotic entry, indicating that inhibition of WEE1 was able to bypass the Dovitinib-induced G_2_ arrest. Interesting, although MK-1775 stimulated mitotic entry equally in cells treated with 1 and 2.5 μM of Dovitinib, the effect on mitotic exit differed markedly. Cell division occurred relatively normally and generated two daughter cells at the lower concentration of Dovitinib. At higher concentration, however, nearly all the cells underwent protracted mitosis and mitotic slippage, generating tetraploid G_1_ cells. These results suggested that even when the Dovitinib-mediated G_2_ DNA damage checkpoint is bypassed, the resulting mitosis is still defective.

**Figure 7:**
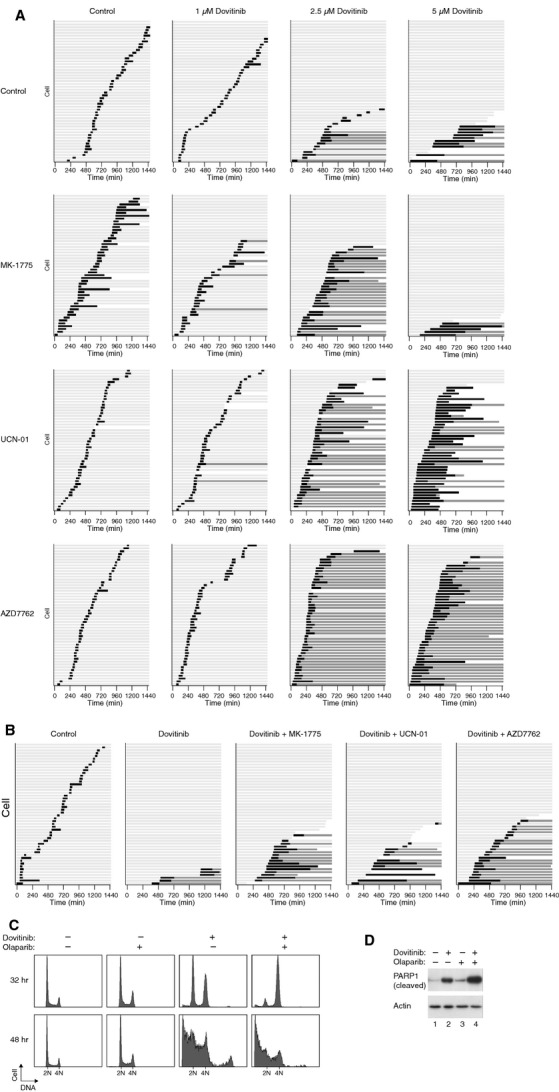
Targeting the DNA damage checkpoint modulates cell cycle arrest and cell death in Dovitinib-treated cells. (A) Inhibition of the G_2_ DNA damage checkpoint abrogates Dovitinib-mediated arrest in HeLa cells. HeLa expressing histone H2B-GFP was incubated with the indicated concentrations of Dovitinib for 16 hrs. Control buffer, MK-1775, UCN-01, or AZD7762 was added just before imaging. Individual cells were then tracked for 24 hrs with time-lapse microscopy. Each horizontal bar represents one cell (*n* = 50). Key is as the same as Figure 2A. (B) Inhibition of the G_2_ DNA damage checkpoint abrogates Dovitinib-mediated arrest in nasopharyngeal carcinoma cells. HONE1 expressing histone H2B-mRFP was incubated with Dovitinib (2.5 μM) for 16 hrs. Control buffer, MK-1775, UCN-01, or AZD7762 was added just before imaging. Individual cells were then tracked for 24 hrs with time-lapse microscopy. Each horizontal bar represents one cell (*n* = 50). Key is as the same as Figure 2A. (C) Inhibition of poly(ADP-ribose) polymerases promotes Dovitinib-mediated cell cycle arrest and cell death. HeLa cells were incubated with 5 μM of Dovitinib and/or Olaparib as indicated. At 32 and 48 hrs, the cells were harvested and analysed with flow cytometry. (D) Inhibition of poly(ADP-ribose) polymerases promotes Dovitinib-mediated apoptosis. HeLa cells were incubated with 5 μM of Dovitinib and/or Olaparib as indicated for 40 hrs. Lysates were prepared and analysed with immunoblotting.

To see if these results could be reproduced in another cell line, HeLa cells were subjected to similar analysis (Fig. 7A). As described above, treatment of HeLa cells with 2.5–5 μM of Dovitinib induced a G_2_ arrest. Inhibition of CHK1 with UCN-01 or AZD7762 uncoupled the arrest and promoted mitosis. As controls, UCN-01 or AZD7762 alone did not affect the cell cycle. Interestingly, although the WEE1 inhibitor MK-1775 could also uncouple the G_2_ arrest induced by 2.5 μM of Dovitinib, it was unable to overcome the checkpoint induced by a higher concentration of Dovitinib. It is possible that at higher concentrations of Dovitinib, inhibition of WEE1 alone could not bypass the checkpoint without activating the CDC25s. As CHK1 lies upstream of both WEE1 and CDC25s, its inhibition could be more effective in uncoupling the checkpoint. Finally, Dovitinib-mediated cell cycle arrest in another cell line, HONE1, was also reduced after inhibition of WEE1 or CHK1 (Fig. 7B).

### Dovitinib-mediated G_2_ arrest can be promoted by inhibition of poly(ADP-ribose) polymerases

Given that Dovitinib could activate the DNA damage checkpoint, we predicted that its effect could be increased if the DNA damage repair pathway is compromised. One of the earliest responses to DNA damage is the binding of poly(ADP-ribose) polymerases (PARPs) to damaged sites. The binding of PARPs to the site of DNA damage activates its catalytic activity and promotes local poly(ADP-ribose)-dependent recruitment of DNA repair enzymes required for efficient DNA repair. Cells were treated with Dovitinib alone or in combination with a poly(ADP-ribose) polymerase inhibitor Olaparib (AZD2281). While a partial G_2_/M arrest was induced in HeLa cells after incubation with 5 μM of Dovitinib for 32 hrs, a more robust arrest was induced by combining Olaparib with Dovitinib (Fig. 7C). This suggested that inhibition of PARP-mediated DNA repair promoted the activation of the G_2_ DNA damage checkpoint by Dovitinib. Longer incubation with Dovitinib induced apoptosis (as indicated by sub-G_1_ population), which also appeared to be promoted by Olaparib (Fig. 7C). This was confirmed by the increase of apoptosis-mediated cleavage of PARP1 (Fig. 7D). These data indicate that inhibition of PARPs increased the Dovitinib-mediated cell cycle arrest and apoptosis, further supporting the idea that Dovitinib could induce DNA damage.

## Discussion

In this study, we demonstrated that Dovitinib promoted cell cycle arrest in both G_2_ and mitosis. A G_2_/M arrest was observed in multiple cell lines, including several from NPC (HONE1, HNE1, CNE2, and C666-1; Fig. 1A), cervical carcinoma HeLa (Fig. 3A), hepatocellular carcinoma Hep3B (Fig. 4A), and several other cell lines from colon and fibroblasts origin (our unpublished data). Using live-cell imaging of histone H2B-GFP-labelled cells, we found that Dovitinib triggered delays in mitosis and G_2_ in a concentration-dependent manner. While interphase (G_2_) arrest was induced after incubation with relatively high concentrations of Dovitinib, a delay in mitosis was also triggered with the lower concentrations (Figs 2 and 3). These cells exhibited a delay in metaphase–anaphase transition and exited mitosis with mitotic slippage or cytokinesis failure. We also noted that different cell lines displayed different sensitivity to Dovitinib. For example, Dovitinib induced extensive apoptosis in Hep3B, which may explain why no mitotic delay was observed (Fig. 4).

Similar to Dovitinib, several small molecule inhibitors of multiple RTKs have been reported to trigger G_2_/M cell cycle arrest. For example, Sorafenib can induce a G_2_/M arrest in cells from hepatocellular carcinoma 36. Foretinib, a VEGFR-2 and c-MET inhibitor, has also been shown to induce a G_2_/M arrest in cells from hepatocellular carcinoma 37 and ovarian cancer 38. However, the underlying mechanisms of these cell cycle arrests are not known.

Why Dovitinib promoted delays in G_2_ or mitosis? It is conceivable that as a non-specific kinase inhibitor, Dovitinib could also target protein kinases involved in cell cycle control. Indeed, the metaphase–anaphase delay caused by Dovitinib (Fig. 3E) was reminiscent to that caused by inhibitors of the mitotic kinase PLK1 39–40. However, Dovitinib does not have high affinity for serine/threonine-specific kinases 7. A more probably explanation is based on the recent finding that Dovitinib could also target topoisomerases 41. Hasinoff *et al*. realized that Dovitinib structurally resembles the DNA minor groove binding dye Hoechst 33258. Because DNA binding compounds are often topoisomerase inhibitors, Hasinoff *et al*. also investigated the ability of Dovitinib to interact with topoisomerases and found that Dovitinib can inhibit the catalytic activity of topoisomerase I and topoisomerase II 41.

Topoisomerase II is able to catenate and decatenate DNA by introducing a transient double-strand break into DNA and then pass a second DNA strand through the break before resealing it. Together with proteins including condensin, Topoisomerase II contributes to the compaction of the chromosome, thereby ensuring normal mitotic chromatin resolution 42. Many topoisomerase inhibitors are well-known anti-cancer drugs. These topoisomerase inhibitors are either classic topoisomerase poisons, which cause DNA strand breaks through stabilization of intermediate cleavable complexes, or true catalytic inhibitors that prevent decatenation and can induce mitotic slippage 43. Hasinoff *et al*. found Dovitinib can stabilize the cleavable complexes and acts as a topoisomerase II poison 41. This is in agreement with our finding that Dovitinib could induce DNA damage as measured by comet assays (Fig. 5D) and γ-H2AX (Fig. 5E and F). Accordingly, Dovitinib could also activate the G_2_ DNA damage checkpoint. Phosphorylation of both CHK1 and CDK1 was promoted by Dovitinib (Fig. 5C). In support of this, the inhibition of CDK1 and prevention of mitosis could be abrogated with CHK1 inhibitors (Figs 6 and 7). Our data are in accordance with the possibility that Dovitinib also could act as a catalytic inhibitor of topoisomerase II, in particularly at relatively lower concentrations, because of the defective mitotic exit (Fig. 3). Hence it is possible that while relatively low concentrations of Dovitinib inactivated topoisomerase II’s catalytic activity, higher concentrations acted as a classic topoisomerase poison and stabilized intermediate cleavable complexes.

Activation of the G_2_ DNA damage checkpoint by Dovitinib has several implications to potential anti-cancer therapies. One possibility is that Dovitinib may be able to act synergistically with other DNA damaging agents. Combined therapy may promote a stronger G_2_ cell cycle arrest than Dovitinib alone without the side effects of using high dosages of drugs. As the Dovitinib-mediated G_2_ arrest could be uncoupled with small inhibitors of CHK1 or WEE1, another possibility is that Dovitinib can be used in combination with reagents that uncouple the checkpoint (Fig. 7A and B). Forcing damaged cells into premature mitosis triggers mitotic catastrophe and has been developed as a strategy for anti-cancer therapies. Along the similar line, inhibition of DNA repair by, for example, inhibitors of poly(ADP-ribose) polymerases, may promote the DNA damage induced Dovitinib (Fig. 7C and D). In this connection, cancer cells that already have defects in the DNA damage checkpoints or DNA repair pathways may be sensitized to Dovitinib in a synthetic lethal manner.

It should be noted, however, that the concentrations of Dovitinib that induced the G_2_ or mitotic delay was relatively high (1–5 μM) compare to that required to inhibit RTKs (IC_50_ <10 nM) 7. In mouse xenograft models, dose levels associated with anti-tumour efficacy are between 10 and 30 mg/kg, with the corresponding C_max_ between 0.4 and 1.9 μM 7. The clinical exposure of Dovitinib is less than that used in animal models. In one study, the C_max_ was found to be between 0.01 and 0.3 μM 44. Whether a G_2_ or mitotic delay could be achievable with the doses of Dovitinib used in clinical trials should be considered.

In conclusion, Dovitinib induced mitotic exit defects and DNA damage, resulting in mitotic slippage and activation of the G_2_ DNA damage checkpoint, respectively. These results suggest additional directions for use of Dovitinib.

## References

[b1] Lemmon MA, Schlessinger J (2010). Cell signaling by receptor tyrosine kinases. Cell.

[b2] Krause DS, Van Etten RA (2005). Tyrosine kinases as targets for cancer therapy. N Engl J Med.

[b3] Petrelli A, Giordano S (2008). From single- to multi-target drugs in cancer therapy: when aspecificity becomes an advantage. Curr Med Chem.

[b4] Luo J, Solimini NL, Elledge SJ (2009). Principles of cancer therapy: oncogene and non-oncogene addiction. Cell.

[b5] Trudel S, Li ZH, Wei E (2005). CHIR-258, a novel, multitargeted tyrosine kinase inhibitor for the potential treatment of t(4;14) multiple myeloma. Blood.

[b6] Lopes de Menezes DE, Peng J, Garrett EN (2005). CHIR-258: a potent inhibitor of FLT3 kinase in experimental tumor xenograft models of human acute myelogenous leukemia. Clin Cancer Res.

[b7] Lee SH, Lopes de Menezes D, Vora J (2005). *In vivo* target modulation and biological activity of CHIR-258, a multitargeted growth factor receptor kinase inhibitor, in colon cancer models. Clin Cancer Res.

[b8] Taeger J, Moser C, Hellerbrand C (2011). Targeting FGFR/PDGFR/VEGFR impairs tumor growth, angiogenesis, and metastasis by effects on tumor cells, endothelial cells, and pericytes in pancreatic cancer. Mol Cancer Ther.

[b9] Huynh H, Chow PK, Tai WM (2012). Dovitinib demonstrates antitumor and antimetastatic activities in xenograft models of hepatocellular carcinoma. J Hepatol.

[b10] Tai WT, Cheng AL, Shiau CW (2012). Dovitinib induces apoptosis and overcomes sorafenib resistance in hepatocellular carcinoma through SHP-1-mediated inhibition of STAT3. Mol Cancer Ther.

[b11] Sivanand S, Pena-Llopis S, Zhao H (2012). A validated tumorgraft model reveals activity of dovitinib against renal cell carcinoma. Sci Transl Med.

[b12] Lamont FR, Tomlinson DC, Cooper PA (2011). Small molecule FGF receptor inhibitors block FGFR-dependent urothelial carcinoma growth *in vitro* and *in vivo*. Br J Cancer.

[b13] Azab AK, Azab F, Quang P (2011). FGFR3 is overexpressed waldenstrom macroglobulinemia and its inhibition by Dovitinib induces apoptosis and overcomes stroma-induced proliferation. Clin Cancer Res.

[b14] Yam CH, Siu WY, Lau A (2000). Degradation of cyclin A does not require its phosphorylation by CDC2 and cyclin-dependent kinase 2. J Biol Chem.

[b15] Cheung ST, Huang DP, Hui AB (1999). Nasopharyngeal carcinoma cell line (C666-1) consistently harbouring Epstein-Barr virus. Int J Cancer.

[b16] Sizhong Z, Xiukung G, Yi Z (1983). Cytogenetic studies on an epithelial cell line derived from poorly differentiated nasopharyngeal carcinoma. Int J Cancer.

[b17] Glaser R, Zhang HY, Yao KT (1989). Two epithelial tumor cell lines (HNE-1 and HONE-1) latently infected with Epstein-Barr virus that were derived from nasopharyngeal carcinomas. Proc Natl Acad Sci USA.

[b18] Chan YW, Ma HT, Wong W (2008). CDK1 inhibitors antagonize the immediate apoptosis triggered by spindle disruption but promote apoptosis following the subsequent rereplication and abnormal mitosis. Cell Cycle.

[b19] Ma HT, Chan YY, Chen X (2012). Depletion of p31comet protein promotes sensitivity to antimitotic drugs. J Biol Chem.

[b20] Pear WS, Nolan GP, Scott ML (1993). Production of high-titer helper-free retroviruses by transient transfection. Proc Natl Acad Sci USA.

[b21] Siu WY, Arooz T, Poon RY (1999). Differential responses of proliferating *versus* quiescent cells to adriamycin. Exp Cell Res.

[b22] Poon RY, Toyoshima H, Hunter T (1995). Redistribution of the CDK inhibitor p27 between different cyclin.CDK complexes in the mouse fibroblast cell cycle and in cells arrested with lovastatin or ultraviolet irradiation. Mol Biol Cell.

[b23] Singh NP, McCoy MT, Tice RR (1988). A simple technique for quantitation of low levels of DNA damage in individual cells. Exp Cell Res.

[b24] On KF, Chen Y, Ma HT (2011). Determinants of mitotic catastrophe on abrogation of the G2 DNA damage checkpoint by UCN-01. Mol Cancer Ther.

[b25] Tsang YH, Han X, Man WY (2012). Novel functions of the phosphatase SHP2 in the DNA replication and damage checkpoints. PLoS ONE.

[b26] Chan YW, On KF, Chan WM (2008). The kinetics of p53 activation *versus* cyclin E accumulation underlies the relationship between the spindle-assembly checkpoint and the postmitotic checkpoint. J Biol Chem.

[b27] Siu WY, Lau A, Arooz T (2004). Topoisomerase poisons differentially activate DNA damage checkpoints through ataxia-telangiectasia mutated-dependent and -independent mechanisms. Mol Cancer Ther.

[b28] Ganem NJ, Storchova Z, Pellman D (2007). Tetraploidy, aneuploidy and cancer. Curr Opin Genet Dev.

[b29] Cheng Y, Stanbridge EJ, Kong H (2000). A functional investigation of tumor suppressor gene activities in a nasopharyngeal carcinoma cell line HONE1 using a monochromosome transfer approach. Genes Chromosom Cancer.

[b30] Hoppe-Seyler F, Butz K (1993). Repression of endogenous p53 transactivation function in HeLa cervical carcinoma cells by human papillomavirus type 16 E6, human mdm-2, and mutant p53. J Virol.

[b31] Bressac B, Galvin KM, Liang TJ (1990). Abnormal structure and expression of p53 gene in human hepatocellular carcinoma. Proc Natl Acad Sci USA.

[b32] Rogakou EP, Pilch DR, Orr AH (1998). DNA double-stranded breaks induce histone H2AX phosphorylation on serine 139. J Biol Chem.

[b33] Wang Q, Fan S, Eastman A (1996). UCN-01: a potent abrogator of G2 checkpoint function in cancer cells with disrupted p53. J Natl Cancer Inst.

[b34] Zabludoff SD, Deng C, Grondine MR (2008). AZD7762, a novel checkpoint kinase inhibitor, drives checkpoint abrogation and potentiates DNA-targeted therapies. Mol Cancer Ther.

[b35] Mizuarai S, Yamanaka K, Itadani H (2009). Discovery of gene expression-based pharmacodynamic biomarker for a p53 context-specific anti-tumor drug Wee1 inhibitor. Mol Cancer.

[b36] Fernando J, Sancho P, Fernandez-Rodriguez CM (2012). Sorafenib sensitizes hepatocellular carcinoma cells to physiological apoptotic stimuli. J Cell Physiol.

[b37] Huynh H, Ong R, Soo KC (2012). Foretinib demonstrates anti-tumor activity and improves overall survival in preclinical models of hepatocellular carcinoma. Angiogenesis.

[b38] Zillhardt M, Park SM, Romero IL (2011). Foretinib (GSK1363089), an orally available multikinase inhibitor of c-Met and VEGFR-2, blocks proliferation, induces anoikis, and impairs ovarian cancer metastasis. Clin Cancer Res.

[b39] Lenart P, Petronczki M, Steegmaier M (2007). The small-molecule inhibitor BI 2536 reveals novel insights into mitotic roles of polo-like kinase 1. Curr Biol.

[b40] Santamaria A, Neef R, Eberspacher U (2007). Use of the novel Plk1 inhibitor ZK-thiazolidinone to elucidate functions of Plk1 in early and late stages of mitosis. Mol Biol Cell.

[b41] Hasinoff BB, Wu X, Nitiss JL (2012). The anticancer multi-kinase inhibitor dovitinib also targets topoisomerase I and topoisomerase II. Biochem Pharmacol.

[b42] Moser SC, Swedlow JR (2011). How to be a mitotic chromosome. Chromosome Res.

[b43] Cortes F, Pastor N (2003). Induction of endoreduplication by topoisomerase II catalytic inhibitors. Mutagenesis.

[b44] Sarker D, Molife R, Evans TR (2008). A phase I pharmacokinetic and pharmacodynamic study of TKI258, an oral, multitargeted receptor tyrosine kinase inhibitor in patients with advanced solid tumors. Clin Cancer Res.

